# Structure of a Berberine Bridge Enzyme-Like Enzyme with an Active Site Specific to the Plant Family Brassicaceae

**DOI:** 10.1371/journal.pone.0156892

**Published:** 2016-06-08

**Authors:** Bastian Daniel, Silvia Wallner, Barbara Steiner, Gustav Oberdorfer, Prashant Kumar, Eric van der Graaff, Thomas Roitsch, Christoph W. Sensen, Karl Gruber, Peter Macheroux

**Affiliations:** 1 Institute of Biochemistry, Graz University of Technology, Graz, Austria; 2 Institute of Molecular Biosciences, University of Graz, Graz, Austria; 3 Department of Plant and Environmental Sciences, University of Copenhagen, Copenhagen, Denmark; 4 Global Change Research Centre, Czech Globe AS CR, v.v.i., Drásov 470, Cz-664 24 Drásov, Czech Republic; 5 Institute of Molecular Biotechnology, Graz University of Technology, Graz, Austria; Wageningen University, NETHERLANDS

## Abstract

Berberine bridge enzyme-like (BBE-like) proteins form a multigene family (pfam 08031), which is present in plants, fungi and bacteria. They adopt the vanillyl alcohol-oxidase fold and predominantly show bi-covalent tethering of the FAD cofactor to a cysteine and histidine residue, respectively. The *Arabidopsis thaliana* genome was recently shown to contain genes coding for 28 BBE-like proteins, while featuring four distinct active site compositions. We determined the structure of a member of the *At*BBE-like protein family (termed *At*BBE-like 28), which has an active site composition that has not been structurally and biochemically characterized thus far. The most salient and distinguishing features of the active site found in *At*BBE-like 28 are a mono-covalent linkage of a histidine to the 8α-position of the flavin-isoalloxazine ring and the lack of a second covalent linkage to the 6-position, owing to the replacement of a cysteine with a histidine. In addition, the structure reveals the interaction of a glutamic acid (Glu426) with an aspartic acid (Asp369) at the active site, which appear to share a proton. This arrangement leads to the delocalization of a negative charge at the active site that may be exploited for catalysis. The structure also indicates a shift of the position of the isoalloxazine ring in comparison to other members of the BBE-like family. The dioxygen surrogate chloride was found near the C(4a) position of the isoalloxazine ring in the oxygen pocket, pointing to a rapid reoxidation of reduced enzyme by dioxygen. A T-DNA insertional mutant line for *At*BBE-like 28 results in a phenotype, that is characterized by reduced biomass and lower salt stress tolerance. Multiple sequence analysis showed that the active site composition found in *At*BBE-like 28 is only present in the Brassicaceae, suggesting that it plays a specific role in the metabolism of this plant family.

## Introduction

Flavoproteins are a large and diverse family that requires either FMN or FAD as cofactor. The majority of flavoproteins (ca. 90%) act as oxidoreductases in a plethora of biochemical redox processes [1]. Among these, the flavin-dependent oxidoreductases berberine bridge enzyme-like enzymes stand out, because of the bi-covalent attachment of the cofactor to the protein via a histidine and a cysteine [2, 3]. The namesake of this protein family is the berberine bridge enzyme from *Eschscholzia californica* (California poppy), which catalyzes the oxidative ring-closure reaction from (*S*)-reticuline to (*S*)-scoulerine. The C-C-bond formed in this reaction is the so called “berberine bridge”. This reaction marks a branch point in the biosynthesis of isoquinoline alkaloids [4]. As the number of sequenced plant genomes increases constantly, more and more genes encoding BBE-like enzymes have been identified in diverse plant families. Interestingly, most of these plant families are not known to synthesize any alkaloids and thus the role of BBE-like enzymes in these plants remains elusive. Despite this lack of knowledge on the biochemical properties of BBE-like enzymes, they have caught the attention of scientists due to their exceptionally high up-regulation observed during the response to pathogens [5, 6, 7] as well as under salt stress conditions [8, 9]. The BBE-like enzymes are predominantly secreted and have been found to contribute a significant part of the plant cell wall proteome (up to 2.5%) [10]. Moreover, BBE-like enzymes contribute to the expressed secretome during infection by various plant pathogens, suggesting a role in plant-pathogen interactions [11, 12, 13].

Recently, we have shown that two BBE-like enzymes from *Arabidopsis thaliana* oxidize monolignols to their corresponding aldehydes, *i*.*e*. they function as monolignol oxidoreductases [9]. This activity suggests that these enzymes are involved in the manipulation of the extracellular monolignol pool and thereby influence plant cell wall metabolism with as yet unknown implications for lignin formation. A detailed study to reveal the physiological role of the oxidoreductases using knock-out plants is currently under way.

**Fig 1:**
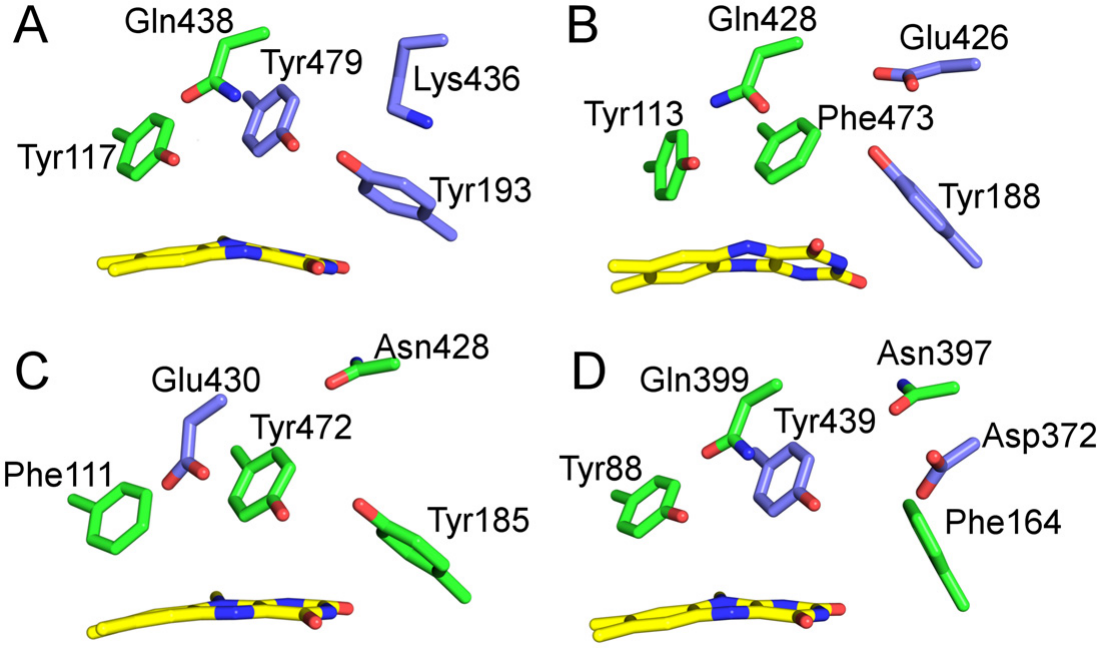
Active site composition of types I-IV (panel A to D) of BBE-like enzymes. The isoalloxazine ring is shown in yellow. Residues forming the active site are shown in green and residues involved in proton abstraction during catalysis are shown in violet. A: Type I: Tyr117 and Gln438 are engaged in a hydrogen bond. Tyr193 acts as catalytic base, it is stabilized in the deprotonated state by Tyr479 and Lys436 (PDB entry 4UD8). B: Type II. Tyr113 and Gln428 are engaged in a hydrogen bond. Tyr188 putatively acts as catalytic base after de-protonation by Glu426 (PDB entry 5D79). C: Type III: Glu430 putatively acts as catalytic base in the active site. The involvement of Tyr472 and Tyr185 is conceivable. A homology model of Q9SA88 based on 4UD8 and prepared with YASARA was used for visualization [14]. D: Type IV: Tyr88 and Gln399 are engaged in a hydrogen bond, Tyr439 acts as catalytic base after de-protonation by Asp372 via a water molecule (PDB entry 4PWC).

Monolignol oxidoreductase activity appears to be associated with a defined composition of the active site, as shown in Fig 1 (panel A). Characteristic features of this active site, designated type I, are Tyr117 and Gln438, engaging in a hydrogen bond as well as Tyr479, Tyr193 and Lys436, forming the catalytic base motif. In this motif, the hydroxyl groups of Tyr479 and Tyr193 point toward each other (oxygen-oxygen distance = 2.4 Å) and Lys436 apparently engages in a cation-π interaction with Tyr193. The type I composition clearly dominates the BBE-like enzyme family in *A*. *thaliana* with 18 out of 28 possessing identical or very similar active sites.

Further inspection of multiple sequence alignments and homology models of BBE-like enzymes revealed the presence of three additional and distinct active site compositions in the remainder of BBE-like enzymes, as shown in Fig 1 (panels B-D, designated type II-IV). In the active site of type II, shown in Fig 1B, the hydrogen bond interaction between Tyr113 and Gln428 is retained, however, the catalytic base motif has substantially changed by exchange of a tyrosine to a phenylalanine and a lysine to a glutamic acid (compare panels A and B). Yet other changes are apparent in the composition of the active sites found in type III and IV (Fig 1C and 1D). The characteristic active site composition described by type II, III and IV are found in 3, 3 and 2 *At*BBE-like enzymes, respectively. Although it appears likely that these BBE-like enzymes also serve as oxidoreductases, neither their substrate spectrum, nor the catalyzed reactions were identified thus far.

Due to the high sequence identity within the BBE-like family, homology models can be valuable tools to support the prediction of the active site composition of structurally uncharacterized members. However, side chain orientations cannot be predicted with the same confidence and thus homology models are not sufficient to determine how residues are oriented in the active site. Therefore, we set out to crystallize a member of the type II BBE-like enzymes in order to pave the way for further studies into their function. Here we report the crystallographic structure of *At*BBE-like 28, a member of the type II BBE-like enzyme sub-family. In addition, we have characterized the enzyme with regard to the oxidative half-reaction and its redox potential and performed initial studies with a homozygous T-DNA knock-out strain, revealing effects on plant growth and salt tolerance. By phylogenetic investigations we have elucidated the appearance of BBE-like proteins with a similar active site as *At*BBE-like 28 in the plant kingdom.

## Results

### Structural characterization of *At*BBE-like 28

**Fig 2:**
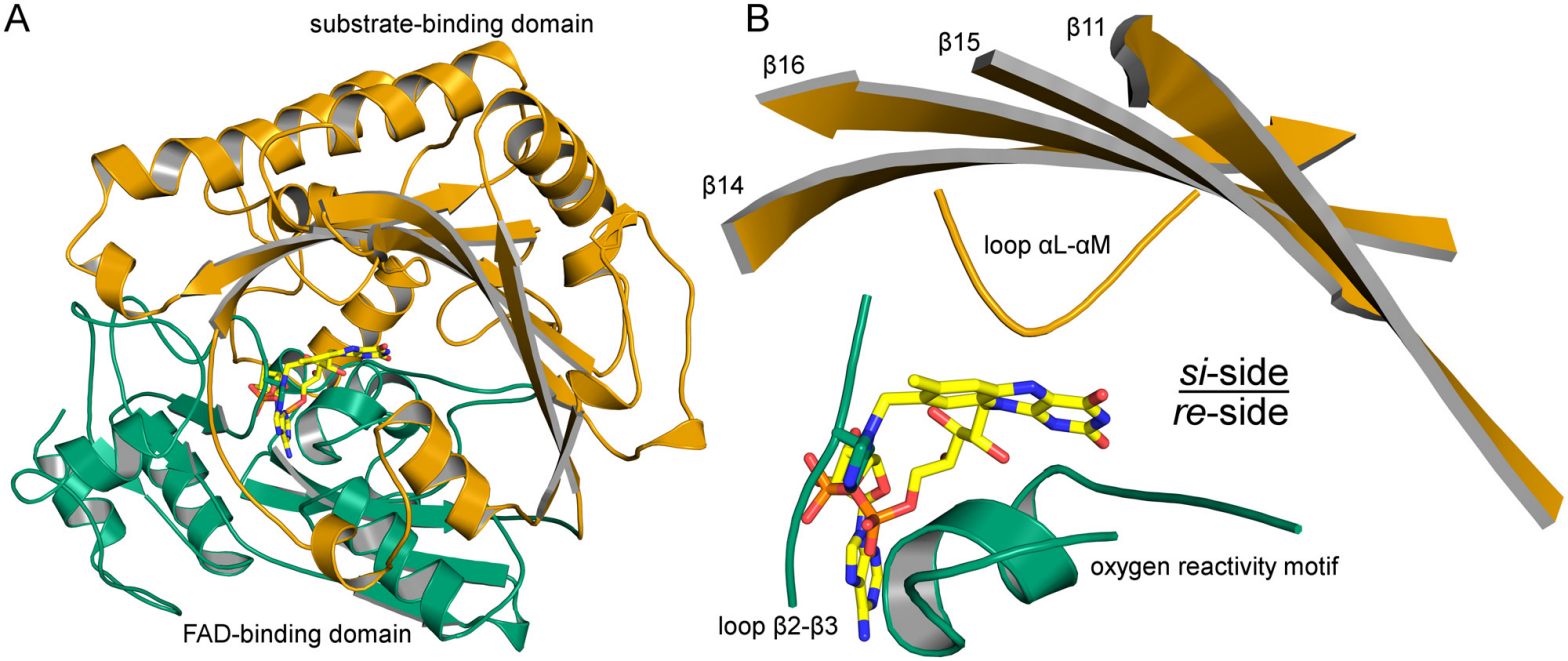
A: Overall topology (A) and active site forming secondary elements (B) of *At*BBE-like 28. A: Green: FAD-binding domain, orange: substrate-binding domain. The active site is formed between the FAD-binding site and a substrate binding domain B: structural elements interacting directly with the isoalloxazine ring of the FAD-cofactor and structural elements harboring residues involved in the formation of the active site.

**Fig 3:**
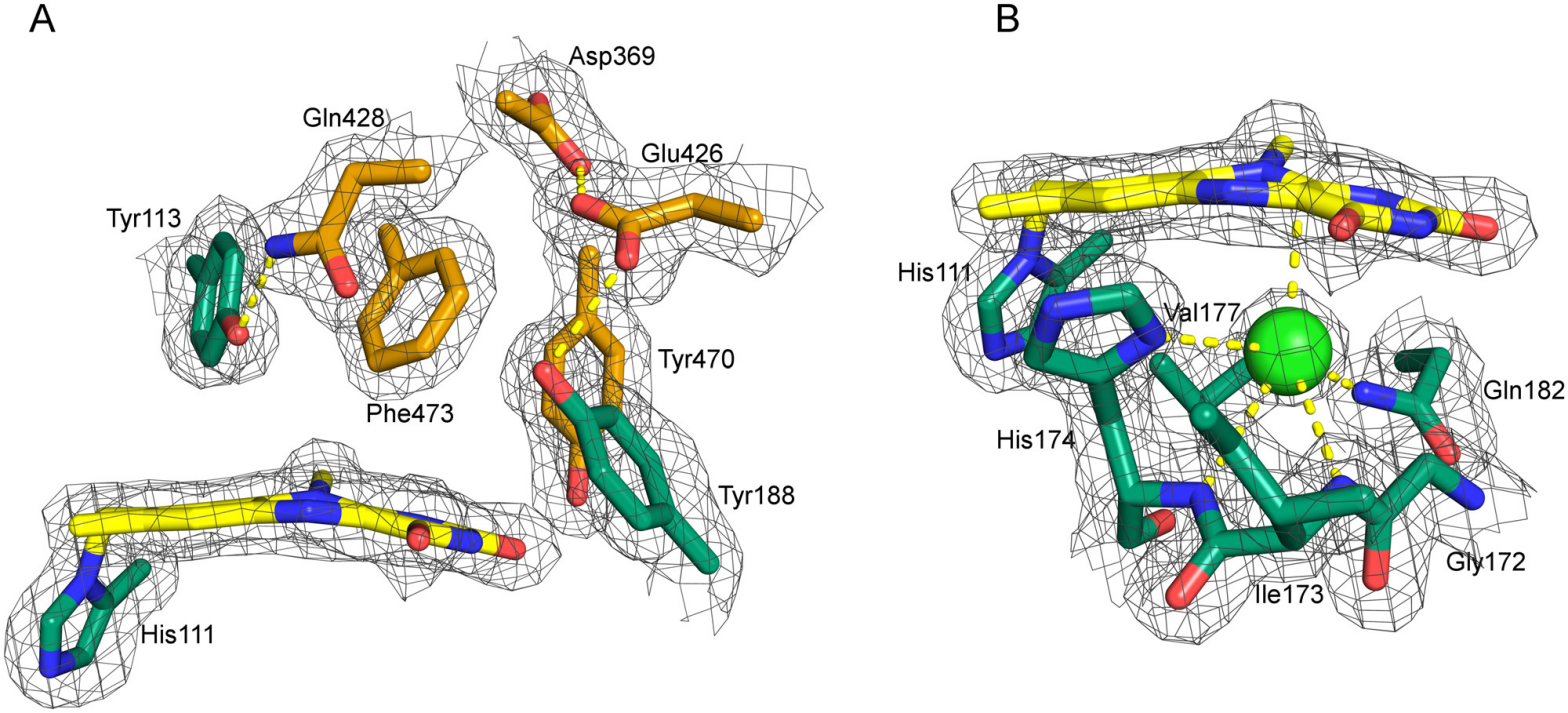
A: Amino acid residues present on the *si*-side (A) and *re*-side (B) of the isoalloxazine ring. A: Residues originating from the FAD- and substrate binding domain are shown in green and orange, respectively. The 2Fo-Fc is shown at 1.5σ. The carboxylate groups of Asp369 and Glu426 are found at a distance of 2.4 Å. The proximity of these residues suggests a shared proton and a negative charge delocalized over both carboxylic acids. Glu426 interacts with Tyr188 that putatively acts as catalytic base after de-protonation by Glu426. B: Residues defining the *re*-side of the isoalloxazine ring. An oxygen pocket is formed by the oxygen reactivity motif (compare Fig 2B). The electron density was interpreted as a chloride ion (light green), which is complexed by the backbone amides of His174 and Ile173, as well as Nπ of His174 and the side chain amide of Gln182.

*At*BBE-like 28 was expressed in *Komagataella pastoris* and purified as reported previously [9]. Crystallization was conducted using the sitting-drop method, yielding crystals diffracting to a resolution of 1.8 Å. The structure was solved using molecular replacement employing the structure of BBE from *Eschscholzia californica* (*Ec*BBE, PDB code 3D2H) as the search template. Like *Ec*BBE, *At*BBE-like 28 adopts the VAO topology, as shown in Fig 2A. The protein consists of two domains: a substrate and a FAD-binding domain, shown in orange and green, respectively (Fig 2). The structural elements directly interacting with the isoalloxazine ring are shown in Fig 2B. Typically, the covalent linkage between the thiol group of a cysteine and the C6-position of the isoalloxazine ring, reported for BBE from *E*. *californica* and *At*BBE-like 15, originate from the oxygen binding motif. In the case of *At*BBE-like 28, the cysteine is replaced by His174, which does not form a covalent bond to the C6-position (Fig 3A and 3B). However, the covalent linkage established by the conserved GGHD motif was clearly seen in the crystal structure (His111 in Fig 3).

As indicated in Fig 2B, the space “above” and “below” the plane of the isoalloxazine ring, *i*.*e*. the *si-* and *re-*side, play different catalytic roles. A catalytic cycle of a flavoprotein can be split into two half reactions. In the resting state, the flavin is oxidized. In the reductive half reaction a putative substrate is oxidized by the flavin, subsequently dissociates from the active site and leaves the flavin in the reduced state. In BBE-like enzymes, this reaction takes place at the *si*-side. In the oxidative half reaction the reduced flavin reacts with an appropriate electron acceptor, *e*.*g*. dioxygen. Enzymes that promote the oxidation of the flavin with dioxygen are classified as an oxidase, enzymes that inhibit this reaction are classified as a dehydrogenase. In the BBE-like family, the oxidation of the flavin by dioxygen is catalyzed at the *re*-side.

The residues defining the composition of the protein on the *si*-side of *At*BBE-like 28 and thereby putatively responsible for the catalysis of the first half reaction are shown in Fig 3A. The flavin adopts a butterfly-bent shape, with an angle at the N5-N10 axis of 6°. A hydrogen bond of 2.6 Å is formed between Tyr113 and Gln428. Tyr470 forms a hydrogen bond to the C(2) = O locus of the isoalloxazine ring. Asp369 and Glu426 are found at a distance of 2.4 Å, indicating that they share a proton. Thus this conformation may stabilize a negative charge, which is delocalized between Asp369 and Glu426 and may be exploited for catalysis.

Direct interactions on the *re*-side of the protein matrix and the isoalloxazine ring are established by the oxygen reactivity motif (Fig 2B). It originates from the FAD binding domain and regulates the reactivity of the enzyme towards dioxygen by sterically controlling access to an oxygen binding pocket near the C(4a)-position of the isoalloxazine ring [15]. Recently, Zafred *et al*. have identified a single residue as the gatekeeper of the oxygen binding pocket: a valine in this position allows the formation of an oxygen-binding pocket, whereas the presence of a leucine occupies the pocket and thus denies access to this site. In *At*BBE-like 28, this position is occupied by Val177 and thus we assume that the pocket is available for oxygen binding. In agreement with this assumption, an electron density in this pocket was clearly detectable and we propose that a chloride ion, acting as an oxygen surrogate, is bound to the pocket (Fig 3B, green sphere). It is complexed by Nπ and the backbone amide nitrogen of His174, with a distance of 3.6 Å and 3.7 Å, respectively. In addition, the chloride ion is complexed by the nitrogen from the backbone amide of Ile173 and side chain amide of Gln182 with a distance of 3.7 Å and 3.3 Å, respectively. The distance between the chloride and the C(4a) of the isoalloxazine ring is 3.1 Å. Dioxygen is thought to attack the reduced flavin to form a C(4a)-peroxy species in the course of the oxidative half reaction.

### Biochemical characterization

**Fig 4:**
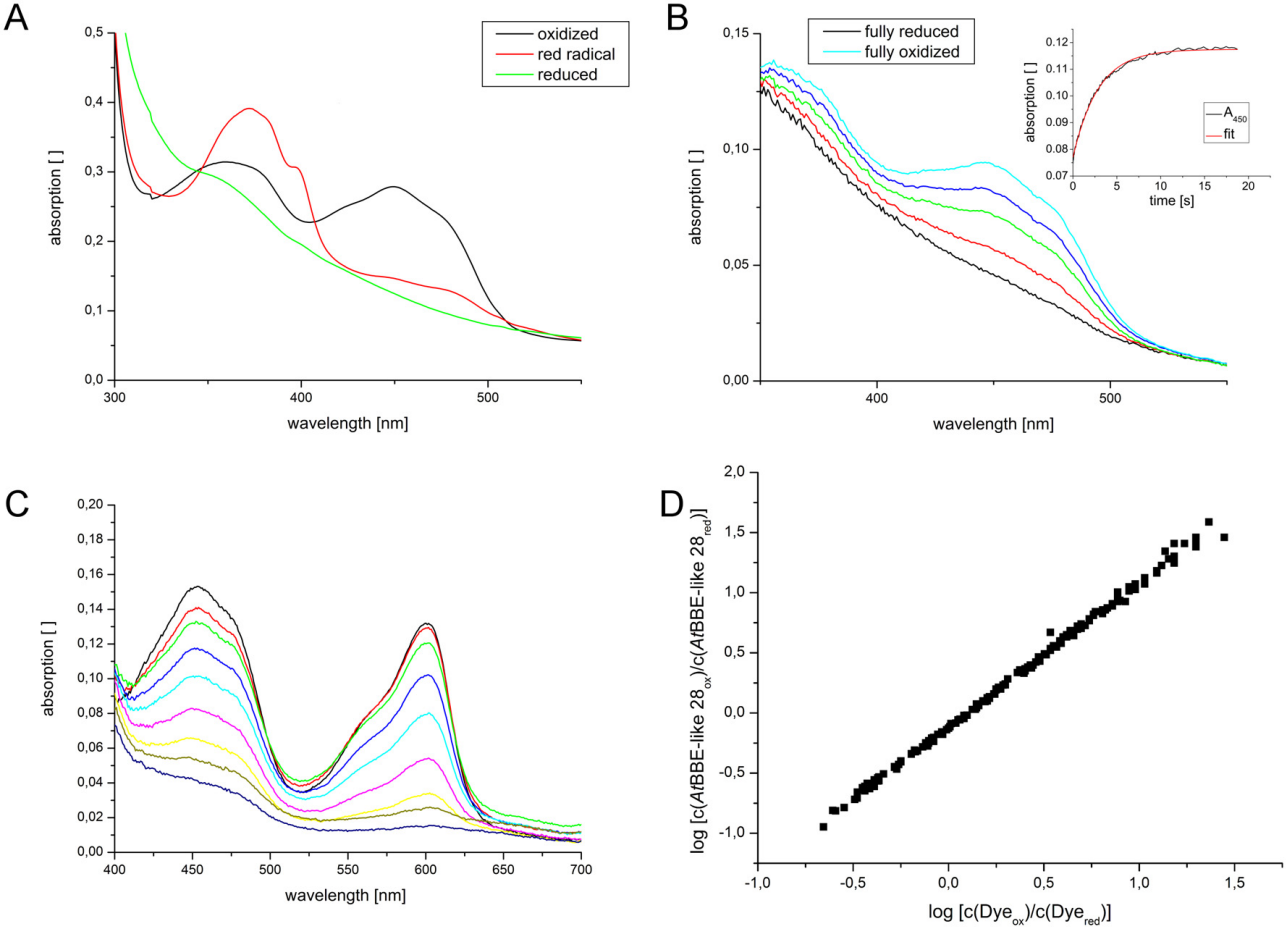
Biochemical characterization of *At*BBE-like 28. A: Photoreduction of A*t*BBE-like 28: The formation of an anionic radical semiquinone species is indicated by the increase of absorption at 370 nm in the course of the reduction B: Selected spectra recorded during the reoxidation of photoreduced *At*BBE-like 28 by oxygen to determine the oxidative rate. Inserted in panel B is the time evolution of the absorbance at 450 nm (black) and a monoexponential fit (red) that was used to determine the oxidative rate. C: Selected spectra of the simultaneous reduction of *At*BBE-like 28 and thionine acetate using the xanthine/xanthine oxidase system as an electron source. D: Double logarithmic plot of the concentration of *At*BBE-like 28 (ox/red) and thionine acetate (ox/red) used for the determination of the redox potential of *At*BBE-like 28.

Recently, we have reported a successful strategy to identify substrates for *At*BBE-like 13 and 15 that involved screening of a chemical compound library based on observed effects on protein thermal stability [9]. In the case of *At*BBE-like 28, this approach was not successful. Similarly, molecular docking of a compound library using our crystallographic structure did not lead to the identification of potential substrates of *At*BBE-like 28. In the absence of a substrate, we thus employed photoreduction according to the method described by Massey *et al*. to gain information on the spectral properties of *At*BBE-28 in its reduced state [16]. Initially, stepwise photoreduction of *At*BBE-like 28 results in an increase of absorption at 370 nm that is characteristic for the anionic (red) flavin semiquinone [17]. Further photoreduction yields the fully reduced dihydroquinone species with a rather featureless absorption spectrum in the visible range. Selected spectra observed during photoreduction are shown in Fig 4A.

The pronounced spectral difference at 450 nm was exploited in additional experiments to investigate the rate of reoxidation of reduced *At*BBE-like 28 by molecular oxygen in a stopped-flow device. Photoreduced enzyme was mixed with air-saturated buffer and the course of re- oxidation of the flavin was followed at 450 nm. Selected spectra recorded during the experiment are shown in Fig 4B. Kinetic analysis of reoxidation yields a bimolecular rate constant of 3040 ± 50 M^-1^ s^-1^ and is thus 12 times faster than the reaction of free FAD with molecular oxygen (250 M^-1^ s^-1^) [18]. Therefore, *At*BBE-like 28 accelerates the rate of reaction with molecular oxygen and can be classified as an oxidase, in agreement with the presence of an oxygen binding pocket near the C(4a)-atom of the isoalloxazine ring, as described in the previous section.

The redox potential is an important parameter to characterize a redox system. In the case of BBE-like enzymes, the bi-covalent cofactor attachment results in a large shift to ca. +130 mV for BBE or +211 mV for the pollen allergen Phl p 4, to give two examples [19, 15]. For the latter this means that the redox potential is shifted by 411 mV compared to that of free FAD (-210 mV) [17]. The redox potential of *At*BBE-like 28 was determined with thionine acetate as reference dye, according to Minnaert *et al*. [20]. Using the xanthine/xanthine oxidase electron delivery system, *At*BBE-like 28 and the dye were simultaneously reduced indicating that the redox systems are in equilibrium during the time course of the experiment. Selected spectra and the double logarithmic plot used for data evaluation are shown in Fig 4C and 4D. A redox potential E° of +61 ± 1 mV was determined for *At*BBE-like 28 at 25°C and pH 7.0.

**Fig 5:**
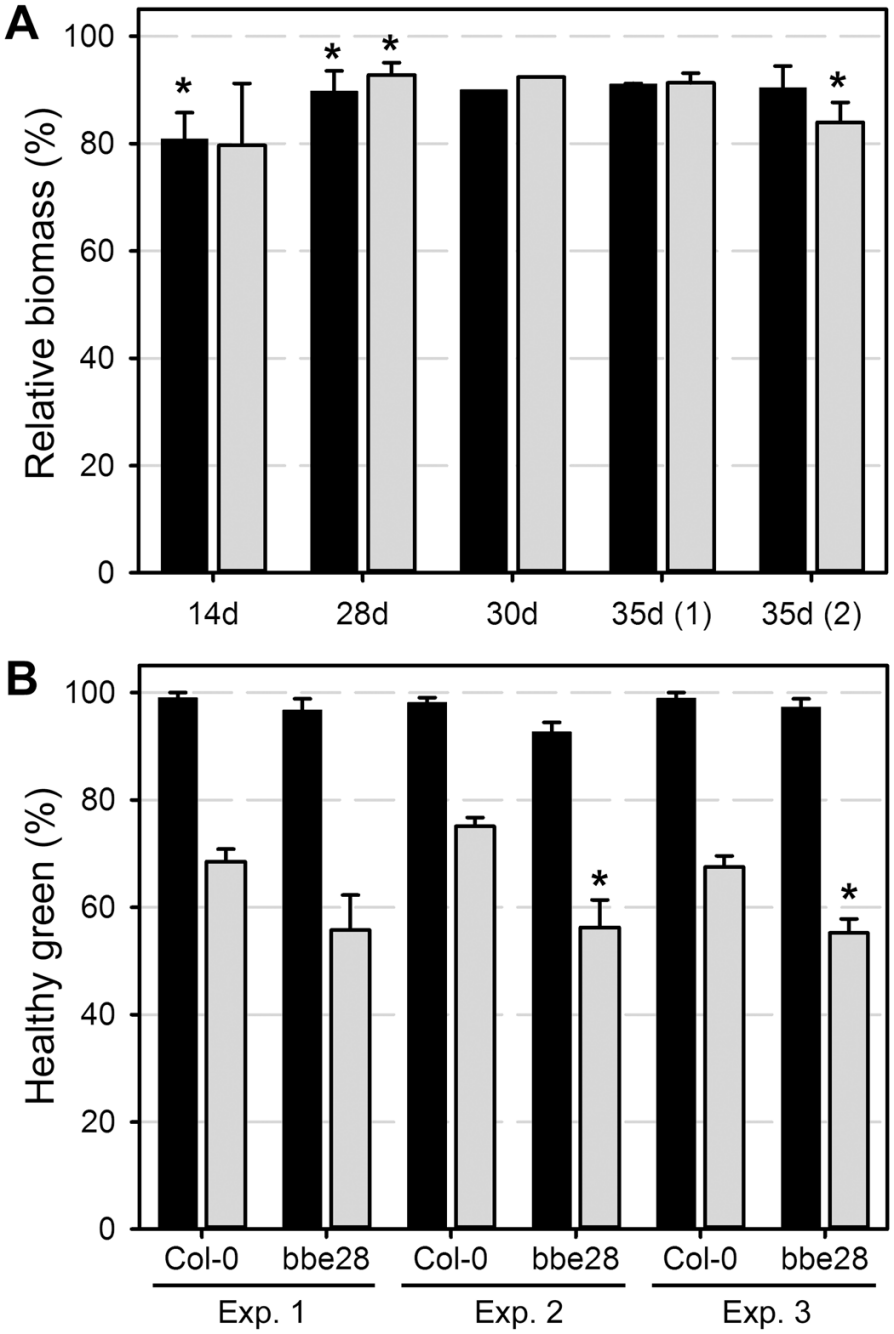
*At*BBE-like 28 loss-of-function affects biomass formation and salt stress tolerance. A: The *bbe28* mutant forms less biomass compared to Col-0, based on fresh weight (FW, black bars) and dry weight (DW, grey bars). B: The *bbe28* mutant is more sensitive to salt stress compared to Col-0. Black bars control medium, grey bars salt stress medium (100 mM NaCl). Average values ± SE are shown, with relative biomass (%) compared to Col-0 in A and percentage of green healthy shoots vs. total number seedlings in B. * indicates the statistically significant difference (Student’s t-test) when compared to Col-0 at p<0.05.

*At*BBE-like 28 functions in biomass formation and salt stress tolerance. To investigate the *in planta* function for *At*BBE-like 28, a T-DNA insertional mutant (*bbe28*) was obtained from the SALK collection with a T-DNA insert in the coding region, representing a putative loss-of-function mutant. The presence of the T-DNA insertion and homozygous *bbe28* mutants were identified by kanamycin selection and PCR analysis. No obvious developmental defects were observed for the homozygous *bbe28* mutant. However, more detailed analysis revealed that *bbe28* produced less biomass (on average 10% reduction) compared to Col-0, based on fresh weight (FW) and dry weight (DW) (Fig 5A) in five independent growth experiments. The *bbe28* seeds germinate at the same rate as Col-0, while *bbe28* plants flower two days later than Col-0 (data not shown). After 30 days of growth, *bbe28* plants produced slightly less leaves (14.8), when compared to Col-0 (15.3), a difference that is much smaller than the 10% reduction in both FW and DW. Based on *in-silico* expression analysis, the expression of many *At*BBE-like family members, is induced under biotic and abiotic stress conditions [21]. Interestingly, the Brassicaceae-specific *AtBBE-like 28* gene is induced by salt stress, specifically in the root. Therefore, we investigated the growth of *bbe28* under (mild) salt stress conditions (100 mM NaCl). Upon direct germination and growth on salt stress medium, both Col-0 and *bbe28* produced lower number of healthy green seedlings (74.3% and 58.4%, respectively) when compared to plants growing on control medium. This increased salt stress sensitivity of *bbe28* was significantly more pronounced compared to the wild type over three replicate experiments (Fig 5B, p = 0.024).

### Sequence comparison and phylogenetic inference

As mentioned in the introduction, *A*. *thaliana* harbors 3 BBE-like enzymes with the active site composition of type II. To investigate how common this particular active site composition is in other plant families we used the sequence of *At*BBE-like 28 to search the genomes of a variety of plants for genes coding for BBE-like enzymes. Our analysis revealed that BBE-like enzymes featuring a type II active site composition can be sub-classified according to their mode of covalent attachment into type IIa and IIb showing monocovalent or bicovalent cofactor attachment, respectively.

**Fig 6:**
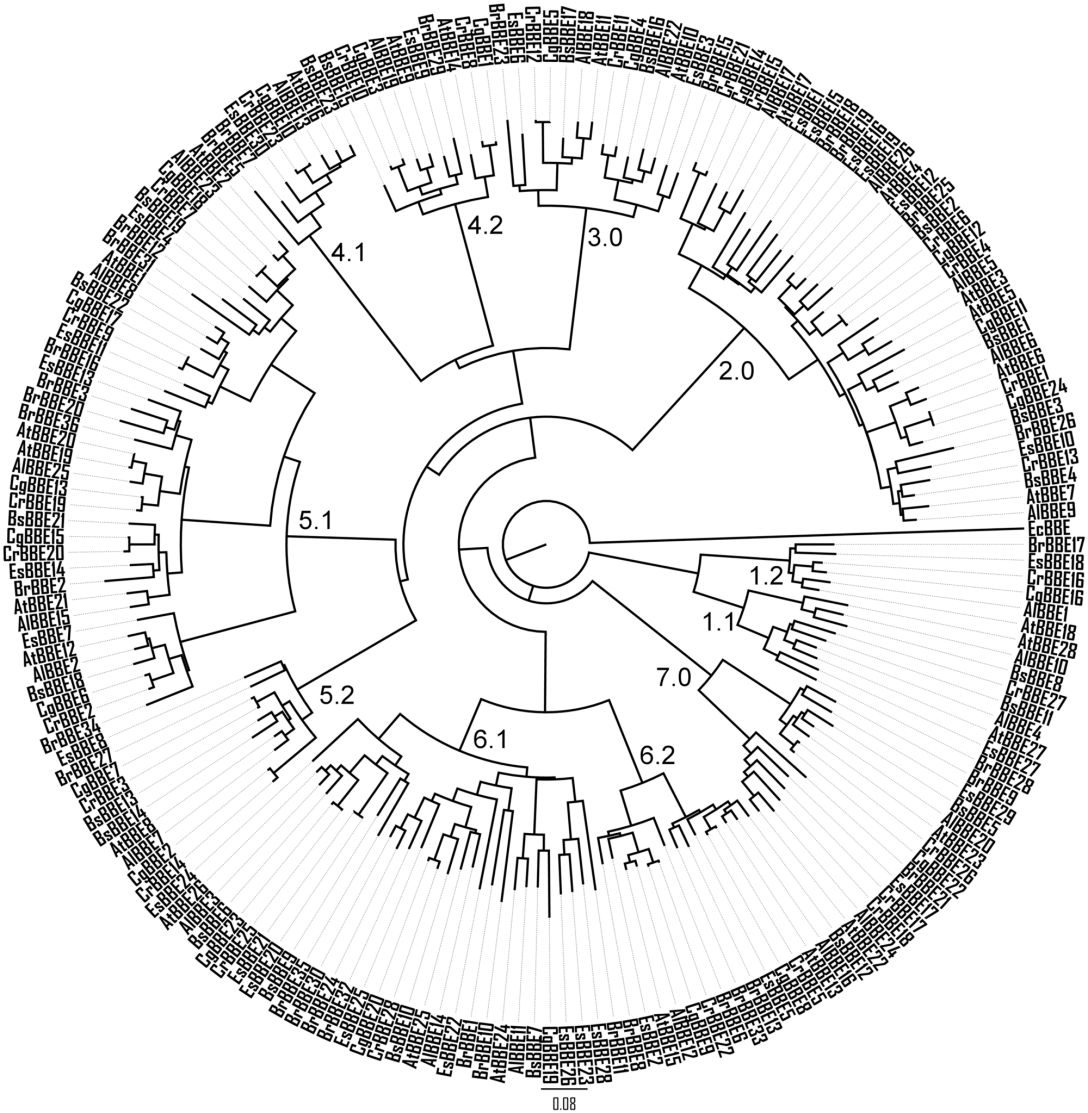
Phylogenetic tree of the BBE-like enzymes within the *Brassicaceae* familiy. Sequences from *Arabidopsis lyrata* (*Al*), *Arabidopsis thaliana* (*At*), *Boechera stricta* (*Bs*), *Brassica rapa* (*Br*), *Capsella grandiflora* (*Cg*), *Capsella rubella* (*Cr*), and *Eutrema salsugineum* (*Es*) were used. The composition of the phylogenetic groups is summarized in Table 1.

Surprisingly, type IIa BBE-like enzymes, such as *At*BBE-like 28, were found exclusively in plant species belonging to the genus Brassicaceae [22]. Therefore, sequences coding for BBE-like enzymes out of the genomes of seven species of this plant family (*i*.*e*. *Arabidopsis lyrata*, *Arabidopsis thaliana*, *Boechera stricta*, *Brassica rapa*, *Capsella grandiflora*, *Capsella rubella*, *and Eutrema salsugineum*) were retrieved from the Phytozome website and aligned with Clustal Omega [23][22]. As shown if Fig 6, a phylogenetic tree was generated using the PHYLIP package version 3.69 [24]. In Table 1, the composition of the phylogenetic groups and the assignment of the distinct active site types (compare Fig 1) are summarized. In all Brassicaceae species except *C*. *grandiflora*, we found at least one enzyme that belongs to type IIa BBE-like enzymes, indicating a widespread occurrence within the Brassicaceae while all other plant families appear to lack BBE-like enzyme with the specific type IIa composition. In our view this may turn out to be an important result in light of the yet unsuccessful search for a cognate substrate of *At*BBE-like 28. The Brassicaceae family is known to possess several specific metabolites, such as the glucosinolates or camalexin, and thus it appears likely that type IIa BBE-like enzymes are involved in specific metabolic (oxidation) processes, which might be confined to the majority of the members of this plant family [25, 26].

**Table 1:** Composition of the phylogenetic groups of the BBE-like enzymes from *Brassicacea* as defined in Fig 6 sorted by species and distribution of the active site types.

Group	Active site type	*At*BBE	*Al*BBE	*Br*BBE	*Bs*BBE	*Cg*BBE	*Cr*BBE	*Es*BBE
1.1	IIa	27, 28	4, 10	28	8, 11		27	27
1.2	IIb	18	1	17		16	16	18
2.0	I	17, 4, 3, 6, 7, 5	5, 6, 9, 17, 26	18, 19, 25, 26	1, 2, 3, 4	11, 12, 14, 24	1, 4, 6, 13, 15, 21	1, 10, 12, 15, 16, 19
3.0	III	10, 11	18, 22	15, 23	16, 17	4, 5	11, 12	3, 6
4.1	III	16	13	5, 7	23	10	23	30
4.2	I	9, 14	19	29	15	1, 3	8, 10	9
5.1	I	1, 2, 12, 19, 20, 21	2, 8, 15, 23, 25	2, 3, 12, 16, 20, 31, 36, 34	18, 19, 22, 21	6, 13, 15, 17, 18	2, 9, 19, 20,	4, 7, 11, 13, 14
5.2	I	8	7	27	13, 14	7, 2	3, 14	8
6.1	I	24, 25, 26	3, 11, 14	1, 10, 11, 24, 30, 32, 35	6, 7, 9, 10	19, 20, 23	25, 28	20, 21, 22, 23, 24, 25, 26, 28
6.2	I	13, 15	12, 16	6, 8, 13, 33	12	8, 9	5, 22	2, 5
7.0	IV	22, 23	20, 24	9, 21	5	22	17, 18, 26	17, 29

### Active site type distribution of BBE-like enzymes

**Fig 7:**
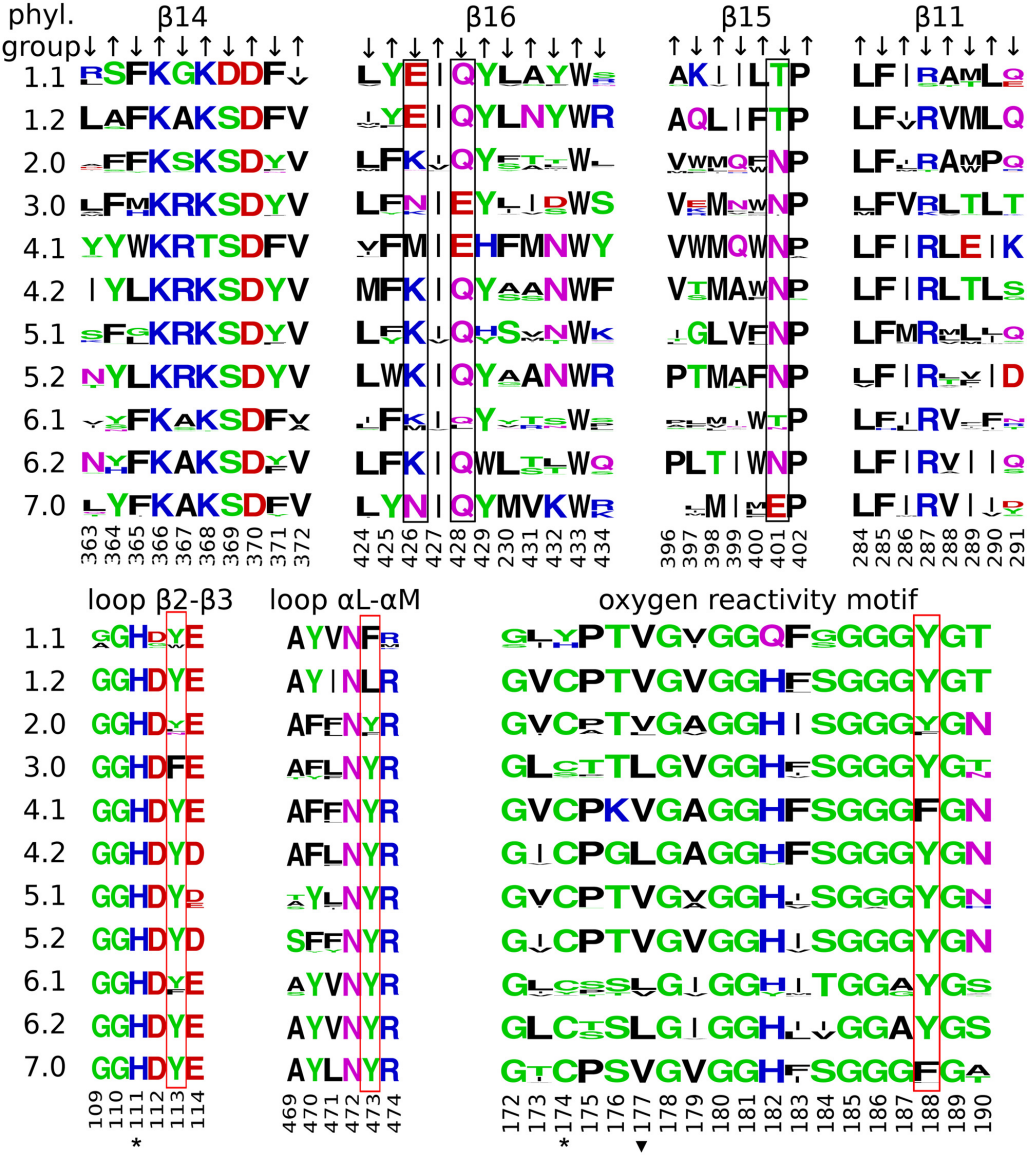
Sequence logos representing the secondary elements directly interacting with the isoalloxazine ring. The numbering is according to the sequence of *At*BBE-like 28. *: site of covalent cofactor attachment, ▼: Gatekeeper residue controlling access to the oxygen pocket; valine is found in oxidases, leucine in dehydrogenases. Black boxes: Variable polar residues putatively involved in catalysis. Red boxes: Variable aromatic residues putatively involved in catalysis. β14, 16, 15 and 11 cover the *si*-side of the isoalloxazine ring. Arrows indicate the orientation of the residues in the β-strands. An upward arrow indicates the residue points towards the α-helices covering the 7-stranded antiparallel β-sheet (compare Fig 2B). These residues are found to be structurally relevant and are predominantly conserved in the overall family (compare β14 positions 366, 368, 370 and β16 position 433). A downward arrow indicates the residue points towards the active site. Residues contributing to the decoration of the active site are conserved within the phylogenetic groups but are variable within the overall family (compare β16 positions 426, 428 and β15 position 401).

Overall, the same phylogenetic groups as described before in the analysis of the *At*BBE-like enzymes were found in the Brassicaceae [9], if reasonable sub groups of the predefined phylogenetic groups are defined according to sequence distances and active site composition. To visualize and compare the active sites of different groups, we generated sequence logos for the amino acids sequences of the pertinent motifs (Fig 2B) as presented in Fig 7. The sequence logos of the active site-forming residues allow the identification of features that are conserved in the overall family or that are specific for a certain sub group. Amino acids belonging to the β-sheets can be differentiated by the orientation of their side chain. Amino acids with a side chain oriented towards the active site are indicated with a downward arrow, while an upwards arrow indicates the opposite orientation of the side chain. In general, the active site of BBE-like enzymes possess a remarkable plasticity that is mainly achieved by the variation of polar contacts at positions 401, 426, and 428 (black boxes in Fig 7) and aromatic residues 113, 188 and 473 (red boxes Fig 7). The mode of covalent cofactor tethering is indicated by amino acid positions 111 and 174 (labeled with * in Fig 7) whereas the reactivity toward oxygen is revealed by inspection of position 172 or 177 (labeled with ▼ in Fig 7).

The active site composition found in *At*BBE-like enzyme 28 is restricted to group 1 and can be differentiated into type a and b, as noted above. Type IIa is present in group 1.1, with the cofactor being attached strictly mono-covalently via His111 (loop β2-β3). In position 174, corresponding to the predominant cysteine responsible for cofactor tethering in other BBE-like enzymes, a histidine or a tyrosine is found (compare Fig 7, group 1.1, Fig 3B). Furthermore, a unique and conserved arrangement is found in type IIa with Asp369 (β14) replacing a highly conserved serine in the rest of the family. This residue is in close contact to Glu426 (β16), which is also invariant in group 1.1. In combination, Asp369 and Glu426 presumably facilitate de-protonation of Tyr188 (oxygen reactivity motif, compare also Fig 3A), which presumably acts as active site base. In group 1.2 a similar arrangement of amino acids is found, with the notable exception of Asp369, hence the catalytic triad appears to be replaced by a dyad. On the other hand, the cofactor in group 1.2 is bi-covalently linked to the protein backbone and Gln182 is replaced by histidine, as is typical for BBE-like enzymes with bicovalent cofactor linkage.

## Discussion

BBE-like enzymes catalyze a broad range of reactions such as two-electron oxidations as reported for *At*BBE-like 15 or four-electron oxidations as shown for Dbv29 [9, 27]. In the case of BBE from *E*. *californica*, substrate oxidation is coupled to a ring closure reaction leading to benzoisoquinoline alkaloids [28]. A similar reaction, which couples substrate oxidation with ring formation, was reported for Δ1-tetrahydrocannabinolic acid synthase [29]. More recently BBE-like enzymes were discovered in the biosynthesis of the ergot alkaloid intermediate chanoclavine I and the indole alkaloid communesin [30, 31]. Although the exact mechanism of the reactions catalyzed by the latter two BBE-like enzymes has not been analyzed in detail yet, the structures of the generated products suggest that a similar coupling of substrate oxidation and ring formation may occur.

### The active site type II

**Fig 8:**
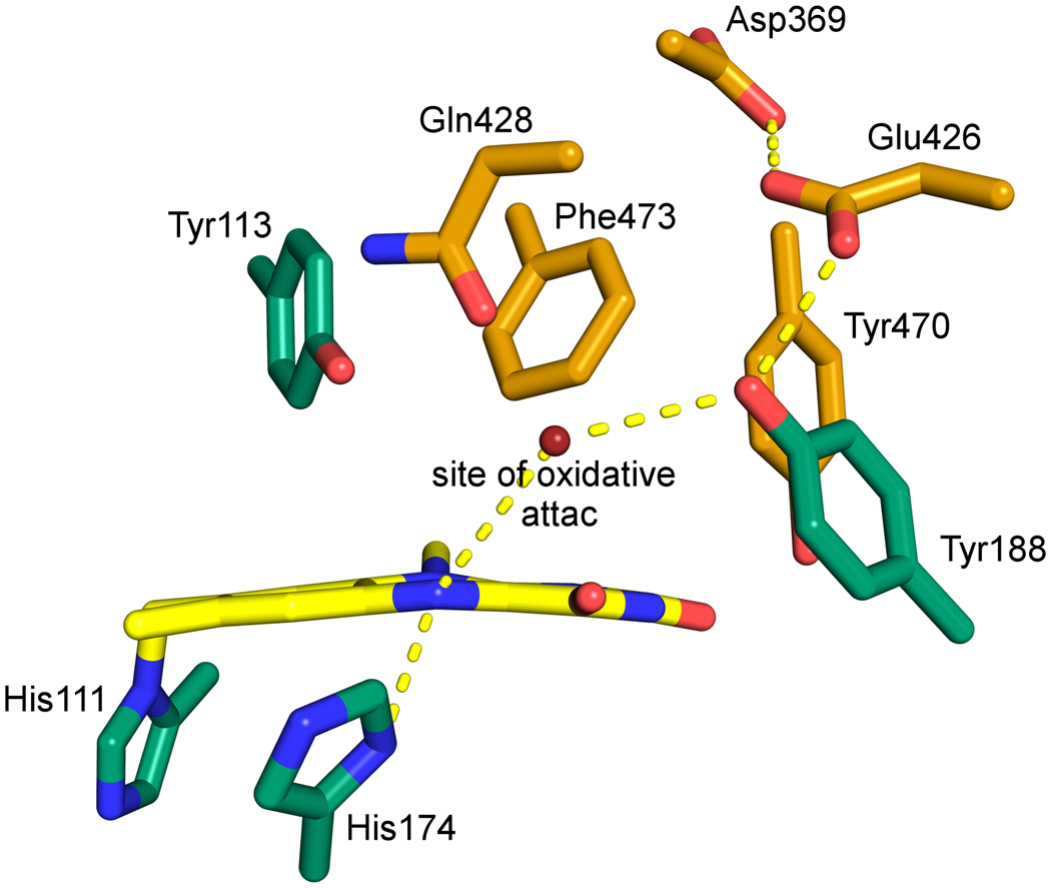
Model of the active site of *At*BBE-like 28 indicating the putative site of oxidative. attack (red sphere). Interactions are represented by dashed yellow lines. One of the unique features of the type IIa active site is the nature and position of the catalytic base, *i*.*e*. Tyr188 that interacts with Glu426 and a monocovalent attachment of the cofactor. In the active site type IIb, nature and position of the catalytic base are conserved (compare Fig 7 1.1 and 1.2) but a bicovalent attachment of the cofactor is to be expected for this active site type. Interestingly the change from a bicovalent to a monocovalent binding mode goes along with the variation of two amino acids of the oxygen reactivity motif. These are the amino acids at position 174 (i.e. cysteine to histidine exchange), responsible for the formation of the covalent bond to C(6) of the isoalloxazine ring and 182 (i.e. histidine to glutamine exchange) (Fig 7). This is perfectly in line with findings reported by Kopacz *et al*. [33] on 6-hydroxy-D-nicotine oxidase, a VAO-type flavoprotein with a monocovalent linkage of a histidine to the 8α-position of the isoalloxazine ring. In this enzyme, bicovalent attachment was achieved by introduction of a cysteine and histidine residue corresponding to position 174 and 182, respectively, indicating that the histidine residue plays a crucial role in the formation of the sulfur-carbon bond [33].

Despite the observed structural differences of the cognate substrates, all BBE-like enzymes share the vanillyl oxidase (VAO) fold. The ability to accept different substrates and catalyze regio- and stereospecific cyclization reactions is controlled by the specific composition of amino acid residues in the active sites. *At*BBE-like 28 has a unique composition of the active site that has not been characterized so far. As the substrate of *At*BBE-like 28 is still unknown, it is difficult to assign distinct roles to the residues in the active site. Fraaije *et al*. have reported some recurrent features present in flavoenzymes that are carrying out an oxidation reaction [32]. According to their analysis, the position of the oxidized atom relative to the flavin ring is conserved (“site of oxidative attack”, see Fig 8). Furthermore, flavin dependent oxidoreductases have a hydrogen bond donor to N(5), a catalytic base to activate the substrate and possess a redox potential above +25 mV. As shown in Fig 8, *At*BBE-like 28 features a hydrogen bond donor to N(5) (Nπ of His174) and a catalytic base to initiate substrate oxidation (Tyr188) and hence shares the hallmarks of a flavoprotein oxidase, as proposed by Fraaije and coworkers [32]. An additional interesting feature of the active site is the proton-relay established between the putative catalytic base, Tyr188, and the neighboring acidic side chains of Glu426 and Asp369, which presumably activate Tyr188 to facilitate proton abstraction. The experimentally determined redox potential of *At*BBE-like 28 of +61 ± 1 mV is also in accordance with a potential function as an oxidase.

### Oxygen reactivity of *At*BBE-like 28

The oxidative half reaction of BBE-like enzymes was recently analyzed in more detail demonstrating that two amino acids fulfill the function of gatekeeper residues in controlling oxygen reactivity [15, 34]. Specifically, the presence of an “oxygen pocket” on the *re*-side of the isoalloxazine ring in direct vicinity of the reactive C(4a)-atom promotes the reaction between dioxygen and reduced flavin in a similar manner as the “oxyanion hole” stabilizes the transition state in hydrolases [15]. This oxygen pocket is created by the oxygen reactivity motif (see Figs 2B and 3B). The access to the oxygen pocket is controlled by amino acids in position 172 and 177. The amino acid glycine in position 172 will allow oxygen to enter while alanine will deny access. Likewise access to the oxygen pocket can be controlled by the size of the side chain of the amino acid in position 177 [34]. The presence of valine in position 177 creates enough space for oxygen to bind in the oxygen pocket, whereas the presence of leucine occupies the oxygen pocket and hence oxygen cannot react with the C(4a)-atom to oxidize the reduced isoalloxazine ring. Thus oxidases and dehydrogenases typically possess either valine or leucine in this position. In *At*BBE-like 28 the gatekeeper positions are occupied by residues typically found in an oxidase, *i*.*e*. glycine and valine. The apparent oxidative rate of 3040 ± 50 M^-1^ s^-1^ is surprisingly small for an oxidase although it is still two to three orders of magnitude larger than the rates found for dehydrogenases of the BBE-like enzyme family [9, 15]. In principle, reduced flavoenzymes may be oxidized by formation of a binary complex with dioxygen after the dissociation of the product (as in a “ping-pong”-mechanism) or the formation of a ternary complex involving the oxidized product [35]. In the case of *At*BBE-like 28 the oxidative and reductive half reaction are spatially separated by the isoalloxazine ring and therefore formation of a ternary complex is conceivable. Thus the rate of reoxidation of reduced *At*BBE28 by dioxygen may also be affected by the presence of product, *i*.*e*. differs from the rate of reoxidation in the binary complex.

### Comparison of *At*BBE-like to another BBE-like protein: Phl p 4

**Fig 9:**
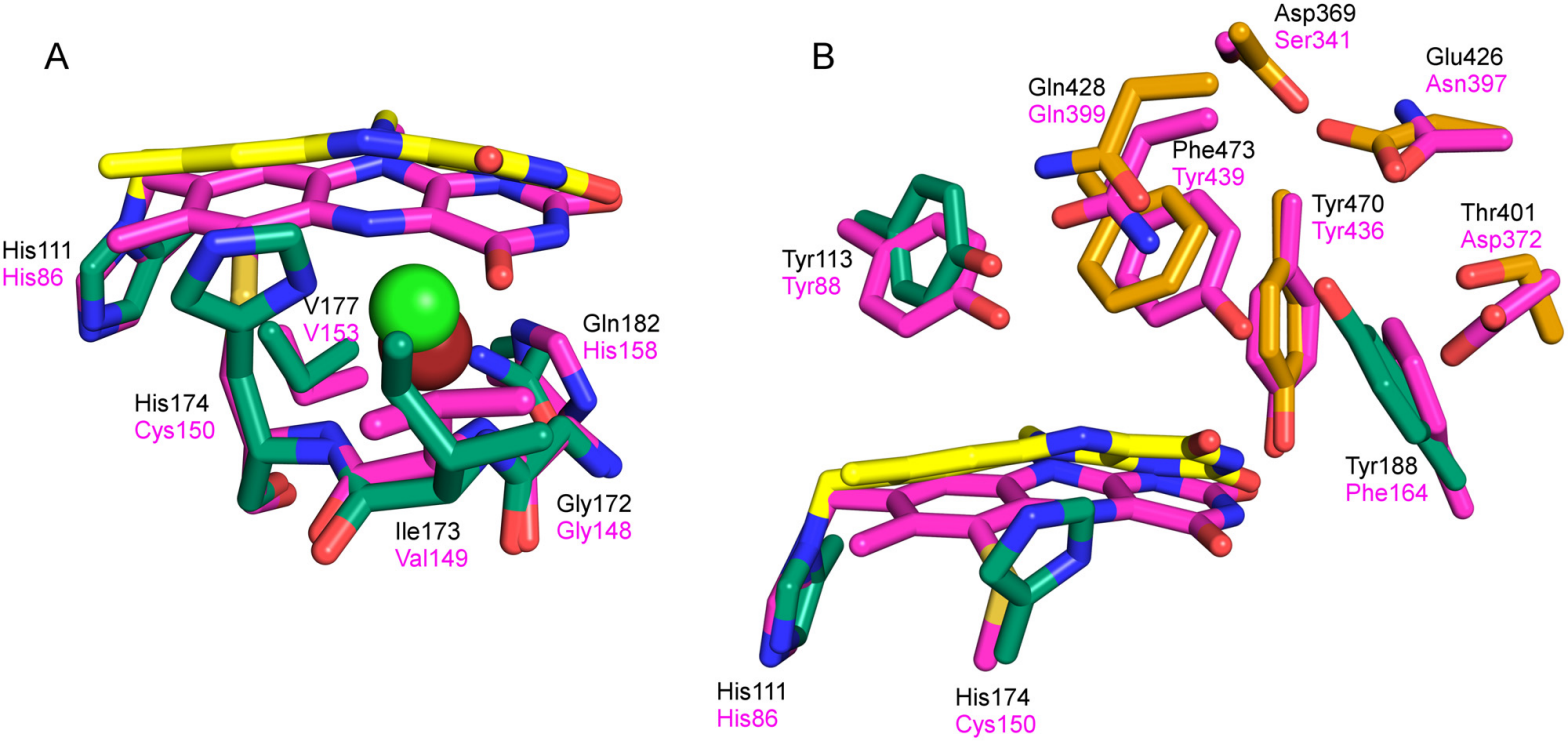
Overlay of *At*BBE-like 28 and Phl p 4 I153V N158H. A: *re*-side: *At*BBE-like 28 is shown in green and yellow, Phl p4 (PDB: 4PWC) is shown in magenta. The chloride ion embedded in *At*BBE-like 28 is shown in green, bromide from Phl p 4 is shown in red. The oxygen pocket is highly conserved and in both structures occupied by oxygen surrogating halide ions. In Phl p4 the halide ion is complexed by the nitrogen of the peptide bond between Cys150 and Val149 and the nitrogen of the peptide bond between Val149 and Gly148. In *At*BBE-like 28 the halide ion is complexed by the corresponding residues, additionally His174 and Gln182 are involved in hydrogen bonds towards the chloride ion. B: *si*-side: The positions of the active site forming residues are conserved. Their nature has been changed, leading to two different active sites embedded in a very similar protein scaffold. Though the position of the isoalloxazine ring is highly conserved in the BBE-like family the C4 C6 axis of the plane N10 C4 C6 has been shifted 32° resulting in a displacement of N5 by 1.6 Å.

Crystallographic studies also demonstrated that dioxygen surrogates, such as halide anions, bind to the oxygen pocket [15]. In the case of *At*BBE-like enzyme 28 we observed an electron density in the oxygen binding pocket that was interpreted as a bound chloride (Fig 3B). An overlay of the previously reported structure of Phl p 4, a pollen allergen from timothy grass with glucose dehydrogenase activity [36], and the structure of *At*BBE-like enzyme 28 is shown in Fig 9. The location of the halide binding site relative to the isoalloxazine ring of the flavin is structurally conserved. Previous studies have also shown that a single amino acid replacement at this position reverses reactivity towards oxygen [15, 9]. In this sense, the sequence logos shown in Fig 7 are useful to predict the reactivity towards oxygen in *At*BBE-like enzymes, *i*.*e*. enzymes with valine in position 177 will behave more like oxidases while those with leucine in position 177 will act like dehydrogenases.

### Analysis of the phenotype of the *At*BBE-like 28 loss-of-function mutant

The putative *At*BBE-like 28 loss-of-function mutant *bbe28* exhibits a reproducible phenotype, both under standard growth and mild salt stress conditions. At a salt concentration of 100 mM, *bbe28* produced significantly less healthy green seedlings when compared to Col-0 indicating that *At*BBE-like 28 plays a role in salt stress tolerance, which would agree with the specific induction by salt stress in the root [21, 37]. Interestingly, under standard growth conditions *bbe28* produced less biomass, both based on FW and DW, respectively. This 10% reduction cannot be explained by a slower development, since *bbe28* formed only slightly fewer leaves in comparison to Col-0 after 30 days of growth. Equally, the *bbe28* mutant showed only a marginal delay in flowering. More detailed analyses will be required to determine the mechanism by which the Brassicaceae-specific *At*BBE-like 28 protein functions in plant growth and salt stress tolerance. Based on the *in-silico* expression data, *At*BBE-like 28 is expressed in the root, specifically in the lateral root cap, endodermis, atrichoblasts and weaker expression in the root xylem. This suggests a function in restricting water transport or minimizing water loss in the root. The weak phenotype associated with the *bbe28* mutant agrees with the fact that among the *At*BBE-like family members only for *At*BBE-like protein 15 a mutant phenotype has been described before [38]. This suggests that detailed phenotyping, combined with biochemical analyses is required to study the *in planta* function for *At*BBE-like protein family members, preferable under specific growth conditions that cause pronounced changes of their respective expression. Importantly, the putative *At*BBE-like 28 function in salt stress deduced from *in silico* expression data, was experimentally confirmed under mild salt stress conditions (Fig 5B) [21, 37].

## Experimental Procedures

All chemicals were purchased at Sigma Aldrich (St. Louis, USA) and were of the highest grade commercially available. Restriction enzymes were obtained from Thermo Fisher Scientific (Waltham, Massachusetts, USA). Ni Sepharose 6 Fast Flow column material was purchased from GE Healthcare (Chalfont St Giles, UK). A synthetic gene coding for *At*BBE28 was from VBC Biotech (Vienna, Austria).

### Cloning and transformation

The protein was expressed using *Komagataella pastoris* as expression host according the EasySelect^™^ *Pichia* Expression Kit provided by Invitrogen (Carlsbad, USA). The gene was adapted to *K*. *pastoris* codon usage and a C-terminal His-tag was added. SignalP was used to identify the native signal sequence of 27 amino acids [39]. The gene lacking the signal sequence was cloned into the pPICZα vector^®^ (Invitrogen), using standard techniques. *K*. *pastoris* strain KM71H was transformed with pPICK-PDI vector harboring the gene for the protein disulfide isomerase from *Saccharomyces cerevisiae*. The modified KM71H strain was transformed with the linearized pPICZα-*At*BBE-like 28 construct. All transformations were done by electroporation. Applicable expression strains were identified using the method proposed by Weis *et al*.[40].

### Expression and purification

Expression was carried out using a BBI CT-2 fermenter (Sartorius AG, Goettingen, Germany) in basal minimal medium as described by Schrittwieser *et al*. [41]. After 96 h of induction the pH was set to 8.0 and the cells were removed by centrifugation at 3000 g at 4°C for 30 min. The supernatant was subsequently filtered through membranes with pore diameter of 0.8 μm, 0.45 μm and 0.22 μM. The volume was reduced from 3 to 0.5 L using a cross flow apparatus (Centramate 500S, Pall Corporation, Dreieich, Germany), equipped with a membrane with a cut-off of 22 kDa (Omega Centramate membrane cassettes, Pall Corporation, Dreieich, Germany). The buffer was changed to 50 mM potassium phosphate pH 7.0 containing 150 mM NaCl and 10 mM imidazole. The supernatant was loaded to a Ni Sepharose^™^ 6 Fast Flow column. The column was washed with 10 column volumes loading buffer, the protein was eluted using 50 mM phosphate buffer containing 150 mM NaCl and 150 mM imidazole. After elution, the column was stripped using 20 mM potassium phosphate buffer pH 7.4 containing 0.5 M NaCl and 50 mM EDTA. A second yellow flavoprotein containing fraction was collected. The purity of the different fractions was checked by SDS-PAGE. Fractions eluted with 50 mM phosphate buffer containing 150 mM NaCl and 150 mM imidazole were concentrated using Amicon Ultra centrifugal filter units (Merck KGaA, Darmstadt, Germany) and loaded on a Superdex 200 gel filtration column using an Äkta system (GE Healthcare, Little Chalfont, United Kingdom). Separation was achieved using 50 mM TRIS/HCl buffer pH 8 containing 150 mM NaCl. Fractions containing *At*BBE-like 28 were concentrated and the buffer was changed to 20 mM TRIS/HCl (buffer A). The protein was loaded to a MonoQ column and eluted applying a stepwise gradient to 100% of a 20 mM TRIS/HCl buffer pH 8 containing 1 M NaCl (buffer B). The flow rate was 1 mL/min, buffer A was kept at 100% for 5 min, then a gradient to 20% buffer B in 30 min was set. 20% buffer B was kept for 10 min, and then a gradient to 100% B was set in 20 min.

### Crystallization and crystal structure determination

The *At*BBE-like 28 fraction collected during column stripping was used for crystallization without further purification steps. After purification *At*BBE-like 28 was stored over night at 4°C in 20 mM potassium phosphate buffer pH 7.4 containing 0.5 M NaCl and 50 mM EDTA. The buffer was changed to 20 mM TRIS/HCl pH 9.0 using a PD-10 desalting column (GE Healthcare, Little Chalfont, United Kingdom) and the protein concentration was adjusted to 20 mg/mL. Crystallization in the optimized condition was performed using the microbatch method, mixing 1 μL of protein and 0.5 μL of 0.1 M HEPES buffer pH 7.0 containing 30% v/v Jeffamine ED 2001 pH 7.0 at 277 K. Tetragonal bipyramidal crystals appeared after streak seeding and grew to a maximum dimension of approximately 300 μm within 14 days. Surplus crystallization buffer was removed by streaking the crystals over the microbatch plate’s surface under oil before flash freezing the crystals in liquid nitrogen without any additional cryo protection. A diffraction data set was collected at 100 K at the beam line BM14 (ESRF, Grenoble, France). The diffraction data were processed using XDS [42]. The structure was solved by molecular replacement using the program Phaser with the structure of *Ec*BBE (PDB ID 3D2H) [43] as search template. Structure refinement was done by repetitive runs of the programs COOT [44] and Phenix [45]. A TLS restrained refinement was included using four TLS groups per chain obtained from the TLSMD webserver [46, 47]. R_free_ values were calculated from 5% randomly chosen reflections excluded from refinement. A summary of data collection, processing and refinement statistics is given in Table 2. Two protein chains were present in the asymmetric unit. Clear electron density was observed for most residues in both chains with the exception of gaps between Tyr37 and Thr43 in chain A and between Cys34 and Val48 in chain B.

**Table 2:** Data collection and refinement statistics.

Wavelength (Å)	0.920
Beamline	ESRF BM14
Resolution range (Å)	15.89–1.849 (1.915–1.849)
Space group	*C*222_1_
Unit cell	127.02 133.13 139.00 90 90 90
Total reflections	376130 (35085)
Unique reflections	93337 (9111)
Multiplicity	4.0 (3.9)
Completeness (%)	93.16 (91.72)
Mean I/sigma(I)	7.22 (2.00)
Wilson B-factor	23.30
R-merge	0.1224 (0.5576)
R-meas	0.1406
CC1/2	0.987 (0.767)
CC*	0.997 (0.932)
R-work	0.1781 (0.2913)
R-free	0.2205 (0.3205)
Number of non-hydrogen atoms	9336
Macromolecules	7927
Ligands	118
Water	1291
Protein residues	994
RMS(bonds)	0.007
RMS(angles)	1.10
Ramachandran favored (%)	95
Ramachandran outliers (%)	0.2
Clashscore	9.98
Average B-factor	33.70
Macromolecules	32.80
Ligands	25.70
Solvent	39.70
PDB code	5D79

### Redox potential determination

The redox potential was determined using the dye-equilibrium method employing the xanthine/xanthine oxidase system as electron source and thionine acetate at the reporter dye (E° = 64 mV) as described by Massey *et al*. [48] Experiments were performed in 50 mM potatassium phosphate buffer pH 7.0 at 25°C. The reaction mixture contained 5 μM methyl viologen as mediator, 15 μM enzyme and xanthine oxidase in catalytic amounts (approximately 2 nM). Solutions with methylviologen, *At*BBE-like 28 and xanthine were mixed under anoxic conditions with solutions of xanthine oxidase and thionine acetate. Reactions were carried out using a stopped flow device SF-61DX2 (TgK Scientific, Bradford-On-Avon, UK) positioned in a glove box (Belle Technology, Weymouth, UK). Spectra were recorded with a KinetaScanT diode array detector MG-6560 (TgK Scientific). One experiment lasted for 50 min, 300 scans were recorded during that time. The potential of *At*BBE-like 28 was calculated from the plot of log ([ox]/[red]) of the protein *versus* log([ox]/[red]) of thionine acetate according to Minnaert *et al*. [20].

### Photoreduction

Photoreduction was carried out according to Massey *et al*. [16]. Reactions were carried out in 50 mM potassium phosphate buffer pH 7.0 at 25°C. Solutions were flushed with nitrogen and stored 1 h in an anaerobic glove box to remove all oxygen. Reaction mixtures containing 20 μM *At*BBE-like 28 and 10 mM EDTA were tightly closed in the glove box and irradiated with a projector (Luminea^™^ 10 W, Pearl GmbH, Buggingen, Germany). Spectra were recorded with a photometer (Specord 205, Analytik Jena, Jena, Germany).

### Determination of the oxidative rate

Photoreduced enzyme was mixed with air saturated buffer in the stopped flow device. Reoxidation of the enzyme was followed at a wavelength of 450 nm. Experiments were performed in 50 mM potatassium phosphate buffer pH 7.0 at 25°C. The oxidative rate was determined using the Kinetic Studio software (TgK Scientific, Bradford-on-Avon, UK)A apparent rate constant was calculated using a monoexponential fitting model.

### Plant growth experiments

*A*. *thaliana* plants (Col-0 ecotype) were grown on half strength MS medium under short day conditions (8 h light) at 21°C (light intensity: 180 μmol m^-2^ s^-1^) in growth cabinets or in soil under long day conditions (16 h light) in a greenhouse at 20–22°C and relative humidity 70% as described in [49]. A T2 T-DNA insertion line was obtained for *At*BBE-like 28 (At5g44440, SALK_007813) from the SALK mutant collection [50]. Homozygous T3 seeds were generated, confirmed by growth of the respective seedlings on kanamycin selection medium and PCR analysis using the primers: bbe28-F 5’-CAAATAACCGATGCACA-3’, bbe28-R 5’-GTCGAGAAAGAAAACCCTAA-3’ and Salk-LBa1 5’-TGGTTCACGTAGTGGGCCATCG-3’. The Col-0 and homozygous *bbe28* seeds employed for the growth experiments were obtained from the respective parental plants that were grown in parallel in soil under greenhouse conditions to ensure an equal seed quality. To determine differences in salt stress tolerance, surface sterilized seeds were germinated and grown on plates with half strength MS medium (control) or salt stress (100mM NaCl) medium for 14 days, three biological replicates, each with three technical replicates and each plate with >30 Col-0 and >30 *bbe28* seeds to minimize variability between plates. For biomass experiments, seeds were germinated and grown on half strength MS medium for 14 days, seedlings were transferred to soil after 14 (28 d) or 16 days (30d) of growth on half strength MS medium and subsequent growth in soil for 14 days, or seeds were directly germinated on soil and grown for 35 days in two independent experiments. The biomass experiments in plates were performed with four technical replicates and 10 Col-0 or *bbe28* plants were weighed per replicate. The biomass experiments in soil were performed with four technical replicates and 12 Col-0 or *bbe28* plants were weighed per replicate, except for the 30d biomass experiment where all 48 plants were weighed in one batch.

### Phylogenetic analysis

Sequences were retrieved from the Phytosome 10.2 website [22]. Clustal omega was used to create a multiple sequence alignment including all *At*BBE-like protein family members using the default parameters [23]. The alignment was manually edited by hand using Jalview [51]. The PHYLIP package (PHYLIP 3.69) was used to create a bootstrapped phylogenetic tree using the programs SEQBOOT, PROTDIST, NEIGHBOR and CONSENSE [24]. We created 1000 Jackknife sub-alignments with SEQBOOT, which were subsequently subjected to a bootstrapped protein-distance analysis. We chose *Ec*BBE (AC39358) as the outgroup sequence. The tree shown in Fig 5 was visualized using Figtree (Tree Fig Drawing Tool, version 1.4.0 by Andrew Rambaut) [52]. The tree including the bootstrap values is shown in the supplement as S1 Fig. The accession codes for the sequences used for the analysis are given in S1 Table.

## Supporting Information

S1 Fig Phylogenetic tree with bootstrap values. The bootstrap values for the phylogenetic tree of BBE-like enzymes from the following species are depicted: *Arabidopsis lyrata* (*Al*), *Arabidopsis thaliana* (*At*), *Boechera stricta* (*Bs*), *Brassica rapa* (*Br*), *Capsella grandiflora* (*Cg*), *Capsella rubella* (*Cr*), and *Eutrema salsugineum* (*Es*).
(TIFF)

S1 Table Accession numbers. Summarized are the accession numbers of BBE-like coding sequences that were used for the phylogenetic analysis and their abbreviations. Genes of the following species are listed: *Arabidopsis lyrata* (*Al*), *Arabidopsis thaliana* (*At*), *Boechera stricta* (*Bs*), *Brassica rapa* (*Br*), *Capsella grandiflora* (*Cg*), *Capsella rubella* (*Cr*), and *Eutrema salsugineum* (*Es*).
(DOCX)
