# Supplementary figures and images for: Structure of a Berberine Bridge Enzyme-Like Enzyme with an Active Site Specific to the Plant Family Brassicaceae
Source: PLoS One. 2016 Jun 8;11(6):e0156892. doi: 10.1371/journal.pone.0156892 (PMC4898691; doi:10.1371/journal.pone.0156892)

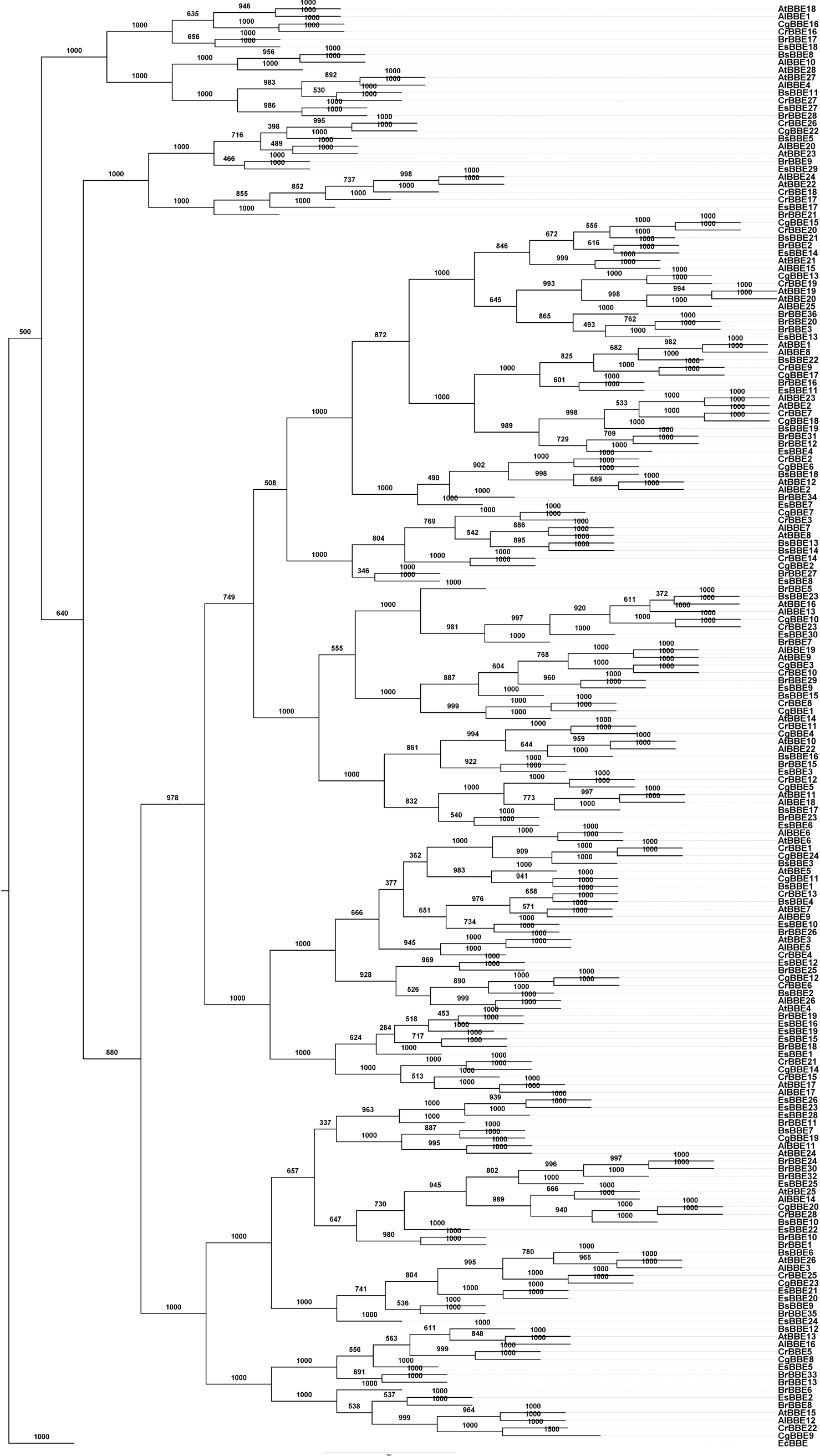

Supplement: S1 Fig — The bootstrap values for the phylogenetic tree of BBE-like enzymes from the following species are depicted: Arabidopsis lyrata (Al), Arabidopsis thaliana (At), Boechera stricta (Bs), Brassica rapa (Br), Capsella grandiflora (Cg), Capsella rubella (Cr), and Eutrema salsugineum (Es). (TIFF) [file pone.0156892.s001.tiff]
