# Supplementary material for: Structure of a Berberine Bridge Enzyme-Like Enzyme with an Active Site Specific to the Plant Family Brassicaceae
Source: PLoS One. 2016 Jun 8;11(6):e0156892. doi: 10.1371/journal.pone.0156892 (PMC4898691; doi:10.1371/journal.pone.0156892)
Supplement: S1 Table — Summarized are the accession numbers of BBE-like coding sequences that were used for the phylogenetic analysis and their abbreviations. Genes of the following species are listed: Arabidopsis lyrata (Al), Arabidopsis thaliana (At), Boechera stricta (Bs), Brassica rapa (Br), Capsella grandiflora (Cg), Capsella rubella (Cr), and Eutrema salsugineum (Es). (DOCX) [file pone.0156892.s002.docx]

| **Gene name** | **Abbreviation** |
| --- | --- |
| AT1G01980.1 | *At*BBE1 |
| AT1G11770.1 | *At*BBE2 |
| AT1G26380.1 | *At*BBE3 |
| AT1G26390.1 | *At*BBE4 |
| AT1G26400.1 | *At*BBE5 |
| AT1G26410.1 | *At*BBE6 |
| AT1G26420.1 | *At*BBE7 |
| AT1G30700.1 | *At*BBE8 |
| AT1G30710.1 | *At*BBE9 |
| AT1G30720.1 | *At*BBE10 |
| AT1G30730.1 | *At*BBE11 |
| AT1G30740.1 | *At*BBE12 |
| AT1G30760.1 | *At*BBE13 |
| AT1G34575.1 | *At*BBE14 |
| AT2G34790.1 | *At*BBE15 |
| AT2G34810.1 | *At*BBE16 |
| AT4G20800.1 | *At*BBE17 |
| AT4G20820.1 | *At*BBE18 |
| AT4G20830.1 | *At*BBE19 |
| AT4G20830.2 | *At*BBE20 |
| AT4G20840.1 | *At*BBE21 |
| AT4G20860.1 | *At*BBE22 |
| AT5G44360.1 | *At*BBE23 |
| AT5G44380.1 | *At*BBE24 |
| AT5G44390.1 | *At*BBE25 |
| AT5G44400.1 | *At*BBE26 |
| AT5G44410.1 | *At*BBE27 |
| AT5G44440.1 | *At*BBE28 |
| Alyrata\|946113\|946113 | *Al*BBE1 |
| Alyrata\|922142\|922142 | *Al*BBE2 |
| Alyrata\|494546\|494546 | *Al*BBE3 |
| Alyrata\|494545\|494545 | *Al*BBE4 |
| Alyrata\|472781\|472781 | *Al*BBE5 |
| Alyrata\|472779\|472779 | *Al*BBE6 |
| Alyrata\|922138\|922138 | *Al*BBE7 |
| Alyrata\|470076\|470076 | *Al*BBE8 |
| Alyrata\|472778\|472778 | *Al*BBE9 |
| Alyrata\|948675\|948675 | *Al*BBE10 |
| Alyrata\|948681\|948681 | *Al*BBE11 |
| Alyrata\|934079\|934079 | *Al*BBE12 |
| Alyrata\|934080\|934080 | *Al*BBE13 |
| Alyrata\|330958\|330958 | *Al*BBE14 |
| Alyrata\|492802\|492802 | *Al*BBE15 |
| Alyrata\|473307\|473307 | *Al*BBE16 |
| Alyrata\|492806\|492806 | *Al*BBE17 |
| Alyrata\|473304\|473304 | *Al*BBE18 |
| Alyrata\|922139\|922139 | *Al*BBE19 |
| Alyrata\|494550\|494550 | *Al*BBE20 |
| Alyrata\|948672\|948672 | *Al*BBE21 |
| Alyrata\|922140\|922140 | *Al*BBE22 |
| Alyrata\|471318\|471318 | *Al*BBE23 |
| Alyrata\|354696\|354696 | *Al*BBE24 |
| Alyrata\|492803\|492803 | *Al*BBE25 |
| Alyrata\|472780\|472780 | *Al*BBE26 |
| Esalsugineum\|Thhalv10017619m.g\|Thhalv10017619m | *Es*BBE1 |
| Esalsugineum\|Thhalv10016496m.g\|Thhalv10016496m | *Es*BBE2 |
| Esalsugineum\|Thhalv10009705m.g\|Thhalv10009705m | *Es*BBE3 |
| Esalsugineum\|Thhalv10007344m.g\|Thhalv10007344m | *Es*BBE4 |
| Esalsugineum\|Thhalv10007327m.g\|Thhalv10007327m | *Es*BBE5 |
| Esalsugineum\|Thhalv10007354m.g\|Thhalv10007354m | *Es*BBE6 |
| Esalsugineum\|Thhalv10007308m.g\|Thhalv10007308m | *Es*BBE7 |
| Esalsugineum\|Thhalv10007341m.g\|Thhalv10007341m | *Es*BBE8 |
| Esalsugineum\|Thhalv10007337m.g\|Thhalv10007337m | *Es*BBE9 |
| Esalsugineum\|Thhalv10007352m.g\|Thhalv10007352m | *Es*BBE10 |
| Esalsugineum\|Thhalv10007313m.g\|Thhalv10007313m | *Es*BBE11 |
| Esalsugineum\|Thhalv10007336m.g\|Thhalv10007336m | *Es*BBE12 |
| Esalsugineum\|Thhalv10024789m.g\|Thhalv10024789m | *Es*BBE13 |
| Esalsugineum\|Thhalv10027426m.g\|Thhalv10027426m | *Es*BBE14 |
| Esalsugineum\|Thhalv10027126m.g\|Thhalv10027126m | *Es*BBE15 |
| Esalsugineum\|Thhalv10027093m.g\|Thhalv10027093m | *Es*BBE16 |
| Esalsugineum\|Thhalv10026881m.g\|Thhalv10026881m | *Es*BBE17 |
| Esalsugineum\|Thhalv10024886m.g\|Thhalv10024886m | *Es*BBE18 |
| Esalsugineum\|Thhalv10027353m.g\|Thhalv10027353m | *Es*BBE19 |
| Esalsugineum\|Thhalv10000843m.g\|Thhalv10000843m | *Es*BBE20 |
| Esalsugineum\|Thhalv10000843m.g\|Thhalv10000842m | *Es*BBE21 |
| Esalsugineum\|Thhalv10000840m.g\|Thhalv10000840m | *Es*BBE22 |
| Esalsugineum\|Thhalv10000851m.g\|Thhalv10000851m | *Es*BBE23 |
| Esalsugineum\|Thhalv10000846m.g\|Thhalv10000846m | *Es*BBE24 |
| Esalsugineum\|Thhalv10001137m.g\|Thhalv10001137m | *Es*BBE25 |
| Esalsugineum\|Thhalv10000841m.g\|Thhalv10000841m | *Es*BBE26 |
| Esalsugineum\|Thhalv10000845m.g\|Thhalv10000845m | *Es*BBE27 |
| Esalsugineum\|Thhalv10000844m.g\|Thhalv10000844m | *Es*BBE28 |
| Esalsugineum\|Thhalv10003322m.g\|Thhalv10003322m | *Es*BBE29 |
| Esalsugineum\|Thhalv10016482m.g\|Thhalv10016482m | *Es*BBE30 |
| Crubella\|Carubv10012287m.g\|Carubv10012287m | *Cr*BBE10 |
| Crubella\|Carubv10011480m.g\|Carubv10011480m | *Cr*BBE11 |
| Crubella\|Carubv10011178m.g\|Carubv10011178m | *Cr*BBE12 |
| Crubella\|Carubv10008831m.g\|Carubv10008831m | *Cr*BBE13 |
| Crubella\|Carubv10008739m.g\|Carubv10008739m | *Cr*BBE14 |
| Crubella\|Carubv10006505m.g\|Carubv10006505m | *Cr*BBE15 |
| Crubella\|Carubv10004576m.g\|Carubv10004576m | *Cr*BBE16 |
| Crubella\|Carubv10007418m.g\|Carubv10007418m | *Cr*BBE17 |
| Crubella\|Carubv10007797m.g\|Carubv10007797m | *Cr*BBE18 |
| Crubella\|Carubv10004466m.g\|Carubv10004466m | *Cr*BBE19 |
| Crubella\|Carubv10007331m.g\|Carubv10007331m | *Cr*BBE20 |
| Crubella\|Carubv10006337m.g\|Carubv10006337m | *Cr*BBE21 |
| Crubella\|Carubv10022971m.g\|Carubv10022971m | *Cr*BBE22 |
| Crubella\|Carubv10022944m.g\|Carubv10022944m | *Cr*BBE23 |
| Crubella\|Carubv10026133m.g\|Carubv10026133m | *Cr*BBE24 |
| Crubella\|Carubv10026198m.g\|Carubv10026198m | *Cr*BBE25 |
| Crubella\|Carubv10027532m.g\|Carubv10027532m | *Cr*BBE26 |
| Crubella\|Carubv10026203m.g\|Carubv10026203m | *Cr*BBE27 |
| Crubella\|Carubv10026190m.g\|Carubv10026190m | *Cr*BBE28 |
| Cgrandiflora\|Cagra.6571s0003\|Cagra.6571s0003.1 | *Cg*BBE1 |
| Cgrandiflora\|Cagra.1508s0130\|Cagra.1508s0130.1 | *Cg*BBE2 |
| Cgrandiflora\|Cagra.1508s0129\|Cagra.1508s0129.1 | *Cg*BBE3 |
| Cgrandiflora\|Cagra.1508s0128\|Cagra.1508s0128.1 | *Cg*BBE4 |
| Cgrandiflora\|Cagra.1508s0127\|Cagra.1508s0127.1 | *Cg*BBE5 |
| Cgrandiflora\|Cagra.1508s0126\|Cagra.1508s0126.1 | *Cg*BBE6 |
| Cgrandiflora\|Cagra.1508s0131\|Cagra.1508s0131.1 | *Cg*BBE7 |
| Cgrandiflora\|Cagra.1508s0123\|Cagra.1508s0123.1 | *Cg*BBE8 |
| Cgrandiflora\|Cagra.3346s0030\|Cagra.3346s0030.1 | *Cg*BBE9 |
| Cgrandiflora\|Cagra.3346s0031\|Cagra.3346s0031.1 | *Cg*BBE10 |
| Cgrandiflora\|Cagra.7100s0001\|Cagra.7100s0001.1 | *Cg*BBE11 |
| Cgrandiflora\|Cagra.7100s0002\|Cagra.7100s0002.1 | *Cg*BBE12 |
| Cgrandiflora\|Cagra.1062s0054\|Cagra.1062s0054.1 | *Cg*BBE13 |
| Cgrandiflora\|Cagra.1062s0058\|Cagra.1062s0058.1 | *Cg*BBE14 |
| Cgrandiflora\|Cagra.1062s0053\|Cagra.1062s0053.1 | *Cg*BBE15 |
| Cgrandiflora\|Cagra.1062s0055\|Cagra.1062s0055.1 | *Cg*BBE16 |
| Cgrandiflora\|Cagra.1968s0134\|Cagra.1968s0134.1 | *Cg*BBE17 |
| Cgrandiflora\|Cagra.0568s0086\|Cagra.0568s0086.1 | *Cg*BBE18 |
| Cgrandiflora\|Cagra.0629s0019\|Cagra.0629s0019.1 | *Cg*BBE19 |
| Cgrandiflora\|Cagra.0629s0018\|Cagra.0629s0018.1 | *Cg*BBE20 |
| Cgrandiflora\|Cagra.0629s0016\|Cagra.0629s0016.1 | *Cg*BBE21 |
| Cgrandiflora\|Cagra.0629s0021\|Cagra.0629s0021.1 | *Cg*BBE22 |
| Cgrandiflora\|Cagra.0629s0017\|Cagra.0629s0017.1 | *Cg*BBE23 |
| Cgrandiflora\|Cagra.7100s0003\|Cagra.7100s0003.1 | *Cg*BBE24 |
| >BrapaFPsc\|Brara.B02784\|Brara.B02784.1 | *Br*BBE1 |
| BrapaFPsc\|Brara.K01808\|Brara.K01808.1 | *Br*BBE2 |
| BrapaFPsc\|Brara.K01807\|Brara.K01807.1 | *Br*BBE3 |
| BrapaFPsc\|Brara.K00501\|Brara.K00501.1 | *Br*BBE4 |
| BrapaFPsc\|Brara.C01758\|Brara.C01758.1 | *Br*BBE5 |
| BrapaFPsc\|Brara.D02108\|Brara.D02108.1 | *Br*BBE6 |
| BrapaFPsc\|Brara.E00947\|Brara.E00947.1 | *Br*BBE7 |
| BrapaFPsc\|Brara.E00948\|Brara.E00948.1 | *Br*BBE8 |
| BrapaFPsc\|Brara.F03747\|Brara.F03747.1 | *Br*BBE9 |
| BrapaFPsc\|Brara.F03748\|Brara.F03748.1 | *Br*BBE10 |
| BrapaFPsc\|Brara.F03744\|Brara.F03744.1 | *Br*BBE11 |
| BrapaFPsc\|Brara.F00789\|Brara.F00789.1 | *Br*BBE12 |
| BrapaFPsc\|Brara.G00508\|Brara.G00508.1 | *Br*BBE13 |
| BrapaFPsc\|Brara.G00588\|Brara.G00588.1 | *Br*BBE14 |
| BrapaFPsc\|Brara.G00589\|Brara.G00589.1 | *Br*BBE15 |
| BrapaFPsc\|Brara.G01552\|Brara.G01552.1 | *Br*BBE16 |
| BrapaFPsc\|Brara.A01126\|Brara.A01126.1 | *Br*BBE17 |
| BrapaFPsc\|Brara.A01123\|Brara.A01123.1 | *Br*BBE18 |
| BrapaFPsc\|Brara.A01124\|Brara.A01124.1 | *Br*BBE19 |
| BrapaFPsc\|Brara.A01128\|Brara.A01128.1 | *Br*BBE20 |
| BrapaFPsc\|Brara.A01152\|Brara.A01152.1 | *Br*BBE21 |
| BrapaFPsc\|Brara.K01265\|Brara.K01265.1 | *Br*BBE22 |
| BrapaFPsc\|Brara.I02784\|Brara.I02784.1 | *Br*BBE23 |
| BrapaFPsc\|Brara.I01845\|Brara.I01845.1 | *Br*BBE24 |
| BrapaFPsc\|Brara.I03113\|Brara.I03113.1 | *Br*BBE25 |
| BrapaFPsc\|Brara.I03114\|Brara.I03114.1 | *Br*BBE26 |
| BrapaFPsc\|Brara.I02786\|Brara.I02786.1 | *Br*BBE27 |
| BrapaFPsc\|Brara.I01846\|Brara.I01846.1 | *Br*BBE28 |
| BrapaFPsc\|Brara.I02785\|Brara.I02785.1 | *Br*BBE29 |
| BrapaFPsc\|Brara.I01844\|Brara.I01844.1 | *Br*BBE30 |
| BrapaFPsc\|Brara.I05161\|Brara.I05161.1 | *Br*BBE31 |
| BrapaFPsc\|Brara.I01843\|Brara.I01843.1 | *Br*BBE32 |
| BrapaFPsc\|Brara.I02779\|Brara.I02779.1 | *Br*BBE33 |
| BrapaFPsc\|Brara.I02783\|Brara.I02783.1 | *Br*BBE34 |
| BrapaFPsc\|Brara.I03596\|Brara.I03596.1 | *Br*BBE35 |
| BrapaFPsc\|Brara.H01046\|Brara.H01046.1 | *Br*BBE36 |
| Bstricta\|Bostr.12659s0359\|Bostr.12659s0359.1 | *Bs*BBE1 |
| Bstricta\|Bostr.12659s0358\|Bostr.12659s0358.1 | *Bs*BBE2 |
| Bstricta\|Bostr.12659s0356\|Bostr.12659s0356.1 | *Bs*BBE3 |
| Bstricta\|Bostr.12659s0355\|Bostr.12659s0355.1 | *Bs*BBE4 |
| Bstricta\|Bostr.3148s0020\|Bostr.3148s0020.1 | *Bs*BBE5 |
| Bstricta\|Bostr.3148s0015\|Bostr.3148s0015.1 | *Bs*BBE6 |
| Bstricta\|Bostr.3148s0017\|Bostr.3148s0017.1 | *Bs*BBE7 |
| Bstricta\|Bostr.3148s0013\|Bostr.3148s0013.1 | *Bs*BBE8 |
| Bstricta\|Bostr.3148s0018\|Bostr.3148s0018.1 | *Bs*BBE9 |
| Bstricta\|Bostr.3148s0016\|Bostr.3148s0016.1 | *Bs*BBE10 |
| Bstricta\|Bostr.3148s0014\|Bostr.3148s0014.1 | *Bs*BBE11 |
| Bstricta\|Bostr.15697s0380\|Bostr.15697s0380.1 | *Bs*BBE12 |
| Bstricta\|Bostr.15697s0370\|Bostr.15697s0370.1 | *Bs*BBE13 |
| Bstricta\|Bostr.15697s0371\|Bostr.15697s0371.1 | *Bs*BBE14 |
| Bstricta\|Bostr.15697s0372\|Bostr.15697s0372.1 | *Bs*BBE15 |
| Bstricta\|Bostr.15697s0373\|Bostr.15697s0373.1 | *Bs*BBE16 |
| Bstricta\|Bostr.15697s0376\|Bostr.15697s0376.1 | *Bs*BBE17 |
| Bstricta\|Bostr.15697s0377\|Bostr.15697s0377.1 | *Bs*BBE18 |
| Bstricta\|Bostr.13671s0285\|Bostr.13671s0285.1 | *Bs*BBE19 |
| Bstricta\|Bostr.10689s0032\|Bostr.10689s0032.1 | *Bs*BBE20 |
| Bstricta\|Bostr.10689s0037\|Bostr.10689s0037.1 | *Bs*BBE21 |
| Bstricta\|Bostr.5325s0118\|Bostr.5325s0118.1 | *Bs*BBE22 |
| Bstricta\|Bostr.23794s0623\|Bostr.23794s0623.1 | *Bs*BBE23 |
